# Bone-Modifying Agents in Early-Stage and Advanced Breast Cancer

**DOI:** 10.1007/s12609-018-0295-6

**Published:** 2018-10-25

**Authors:** Arielle Heeke, Maria Raquel Nunes, Filipa Lynce

**Affiliations:** 1 Levine Cancer Institute, Atrium Health, Charlotte, NC USA; 2 Sidney Kimmel Cancer Center, Johns Hopkins University, Baltimore, MD USA; 3 Georgetown Lombardi Comprehensive Cancer Center, Georgetown University Medical Center, Washington, DC USA; 4 Lombardi Comprehensive Cancer Center, MedStar Georgetown University Hospital, 3800 Reservoir Rd NW, Washington, DC 20057 USA

**Keywords:** Bone-modifying agents, Breast cancer, Denosumab, Zoledronic acid

## Abstract

**Purpose of Review:**

Bone-modifying agents have an important role in the treatment of patients with bone mineral density loss, early-stage breast cancer to reduce risk of recurrence, and metastatic breast cancer with bone involvement. Here we review mechanisms of action of these agents and clinical indications for their use.

**Recent Findings:**

The meta-analysis undertaken by the Early Breast Cancer Trialists’ Collaborative Group showed that the use of bisphosphonates was associated with a decreased risk of breast cancer recurrence.

**Summary:**

The effect of bisphosphonates and receptor activator of nuclear factor kappa-B ligand inhibitors on bone health provides an opportunity to decrease the incidence of skeletal-related events and improve cancer outcomes in certain subsets of patients.

## Introduction

Patients with breast cancer commonly suffer from bone complications. In both localized and advanced disease, accelerated bone mineral density (BMD) loss can occur due to anticancer treatments. Additionally, approximately 70% of patients with metastatic breast cancer will have osseous involvement [1], altering the integrity of their mineralized bone matrix. Strategies to preserve bone health are therefore an important aspect of breast cancer care.

## Mechanism of Action of Bone-Modifying Agents

Osteoclast activation is the main mechanism responsible for both accelerated BMD loss and osteolytic metastases associated with breast cancer. When osteoclasts are activated, multiple signaling cascades are turned on that destabilize the mineralized bone matrix, thereby accelerating BMD loss and creating an environment favorable for tumor cell introduction and overgrowth [2, 3]. Bone-modifying agents, including bisphosphonates (e.g., zoledronic acid) and receptor activator of nuclear factor kappa-B ligand (RANKL) inhibitors (e.g., denosumab), modulate osteoclastic activity to suppress these effects. In preclinical models, bisphosphonate use led to a reduction in the release of bone-derived growth factors [4] and an increase in cytotoxic T cells [5, 6], both of which likely inhibit cancer activity within the bone. Previous studies have also identified increased clearance of disseminated tumor cells, including within the bone marrow, in patients with high-risk, early-stage breast cancer treated with monthly zoledronic acid in addition to chemotherapy, compared to chemotherapy alone [7–9].

Oral and intravenous (IV) bisphosphonates protect bone integrity and density by interrupting hydroxyapatite crystal dissolution during osteoclast-mediated bone resorption. Additionally, bisphosphonates are internalized by endocytosis into osteoclasts leading to apoptosis, thereby providing further protection against osteoclast-mediated resorption in the setting of increased cell death [10]. With second- and third-generation nitrogen-containing bisphosphonates, the enzyme farnesyl pyrophosphate (FPP) is also inhibited, leading to further dysregulation of osteoclast function by creating osteoclast cytoskeletal abnormalities and promoting osteoclast separation from the bone [10]. Denosumab is a fully humanized IgG_2_ monoclonal antibody against RANKL, which activates a receptor expressed on osteoblasts which is a member of the tumor necrosis factor (TNF) family of proteins. Normally, RANKL activates immature osteoclasts to promote osteoclast differentiation, and inhibition of RANKL therefore suppresses this function. Bisphosphonates and RANKL inhibitors may have additional antitumor effects that create a setting less suitable for micrometastatic disease, such as altering tumor vasculature and the immune microenvironment [11, 12]. Notably, levels of RANKL are increased in the presence of bone metastases [13].

## Breast Cancer Treatment Impact on Bone Mineral Density

Several integral therapies used to treat breast cancer are associated with loss of BMD. In premenopausal women treated with chemotherapy, the rate of BMD loss is approximately 3–6% within 12 months of initiating chemotherapy [14–16]. While it is unlikely that chemotherapy is directly toxic to bone structure, chemotherapy-induced amenorrhea leads to BMD loss. Furthermore, premenopausal patients treated with ovarian function suppression experience a 7–11% BMD loss, with partial recovery after therapy is discontinued assuming menses resume [17]. Adjuvant treatment with tamoxifen can also accelerate BMD loss in premenopausal women, with one study citing a 4.6% loss of BMD from baseline in women who remain premenopausal after chemotherapy [18].

In postmenopausal patients, rates of BMD loss are more pronounced. Following treatment with chemotherapy, postmenopausal women experience up to a 10% loss in BMD [19]. In hormone receptor–positive disease, aromatase inhibitor (AI) use further accelerates BMD loss, with partial recovery after the completion of treatment [20, 21]. In comparison to tamoxifen use, which has been associated with BMD gains in postmenopausal women [22], AI therapy is associated with a 40% relative increase in fracture rate [23]. Additionally, 5 years of AI therapy led to the development of osteopenia in 17% of patients treated on the Arimidex, Tamoxifen, Alone, or in Combination (ATAC) study who previously had normal bone density, and osteoporosis in 5% of patients who were osteopenic at trial enrollment [24]. With extended use of an AI, the risk of developing osteoporosis increases further, with new onset osteoporosis developing in 11% of patients treated with extended letrozole compared to 6% treated with placebo on the MA.17R trial [25].

Taking these findings into account, prophylactic bone-modifying agents have been investigated as an adjunct to breast cancer therapies. Several early trials confirmed the role of oral bisphosphonates (e.g., risedronate, clodronate, and ibandronate) in preventing BMD loss, both in pre and postmenopausal patients, when taken for 2 years in conjunction with therapies for early breast cancer [26–28]. More recently, in the Cancer and Leukemia Group B (CALGB) 79809 trial, upfront administration of IV zoledronic acid (4 mg every 3 months for 2 years) in premenopausal women with early breast cancer treated with adjuvant chemotherapy was associated with a significant increase in lumbar spine BMD compared to placebo (1.2% gain versus 6.7% loss) [29]. In postmenopausal patients, the Z-FAST and ZO-FAST trials also assessed upfront zoledronic acid therapy compared to delayed therapy and concluded upfront therapy protected against significant BMD loss [30, 31]. Denosumab has also been shown to reduce BMD loss and aromatase inhibitor-associated fractures in postmenopausal patients [32]. Given these findings, the National Osteoporosis Foundation recommends initiation of bone-modifying agents for postmenopausal women with a history of a fragility fracture, or with osteoporosis identified on a BMD scan defined as a T-score of < − 2.5 [33]. Treatment can also be considered for high-risk patients with T-scores between − 1.0 and − 2.4, with high-risk classification based on their Fracture Risk Assessment Tool (FRAX) score. For most patients, bisphosphonates are favored over denosumab because of the favorable cost and availability of long-term safety data. For patients who are unable to tolerate oral or IV bisphosphonates, denosumab is an alternative option. Additionally, supplemental vitamin D (800 international units daily) and calcium (1200 mg daily) in addition to weight-bearing exercises should be recommended for all patients undergoing breast cancer therapies associated with BMD loss.

## Adjuvant Bone-Modifying Agent Therapy and Breast Cancer–Related Outcomes

In addition to preventing BMD loss, bone-modifying agent therapy should be considered as a component of the adjuvant treatment plan in postmenopausal patients with early-stage breast cancer. In one of the first, large studies of adjuvant bisphosphonate therapy, 302 patients with early breast cancer were selected based on the presence of disseminated tumor cells (DTC) in the bone marrow and were randomly assigned to receive or not oral clodronate 1600 mg daily for 2 years [34]. The primary endpoints were incidence and number of new bony and visceral metastases and the length of time to their appearance. After a median follow-up of 8.5 years, 20.4% of patients who received clodronate were deceased compared to 40.7% in the control group (*p* = 0.049) [35]. However, no significant differences in the incidence of metastases or DFS were appreciated. More recently, adjuvant oral clodronate 1600 mg daily was studied in the National Surgical Adjuvant Breast and Bowel Project (NSABP) B-34 trial, where 3311 patients were randomized to receive bisphosphonate therapy or placebo for 3 years. After a median follow-up of 90.7 months, subgroup analyses identified an improved recurrence-free interval (hazard ratio [HR] 0.75, *p* = 0.045), bone metastasis-free interval (HR 0.62, *p* = 0.027), and non-bone metastasis-free interval (HR 0.63, *p* = 0.014) in postmenopausal patients with no differences in OS [36]. In the German Adjuvant Intergroup Node-Positive (GAIN) study, 3023 node-positive early breast cancer patients were randomized to receive either ibandronate 50 mg by mouth daily or placebo for 2 years; overall results were negative but a trend toward improved DFS was seen in postmenopausal patients who received ibandronate (HR 0.81, *p* = 0.039) [37].

While oral bisphosphonates were chosen in the initial trials, IV formulations are more potent and have been included in the majority of contemporary studies. The ZO-FAST trial, for example, assessed immediate versus delayed adjuvant IV zoledronic acid for 5 years in postmenopausal women, defining indications for delayed use as initiation after a fracture or on-study BMD decrease. Immediate use was associated with improved DFS after a median follow-up of 60 months (HR 0.66, *p* = 0.0375), with fewer local and distant recurrences [31]. In the ABCSG-12 trial, after a median follow-up of 76 months, premenopausal patients with hormone receptor–positive early breast cancer treated with zoledronic acid 4 mg every 6 months for 3 years in addition to ovarian function suppression and endocrine therapy achieved improved OS (HR 0.59, *p* = 0.027) and DFS (HR 0.73, *p* = 0.022) [38, 39]. In this study, the benefit was most pronounced in patients older than age 40. In the AZURE trial, the benefit from 5 years of adjuvant zoledronic acid was again only appreciated in an older population, and specifically in women who were postmenopausal for at least 5 years before enrollment, who had improved OS (HR 0.81, 95% CI (0·63–1·04)) compared to those for whom fewer than 5 years had passed since menopause (HR 1.04, 95% CI (0.86–1.25)) [40, 41]. The benefits of adjuvant bisphosphonate therapy in postmenopausal women with early breast cancer were not reproduced in the Z-FAST study, with a similar DFS and OS seen associated with immediate and delayed use of zoledronic acid for 5 years [42]. In the aforementioned trials for which subgroup analyses included an evaluation of outcomes based on hormone receptor status, no significant differences were appreciated.

To evaluate the potential magnitude of benefit of adjuvant bisphosphonate therapy in patients with early-stage breast cancer, a large meta-analysis of 18,766 individual patient data was carried out by the Early Breast Cancer Trialists’ Collaborative Group (EBCTCG) [43^••^]. Participants were either postmenopausal or premenopausal treated with ovarian function suppression. There was a wide range of the type of bisphosphonate administered and the duration of treatment. The majority of patients included received adjuvant chemotherapy (83%). The study concluded that bisphosphonate therapy was overall associated with a statistically significant improvement in distant recurrence (risk ratio [RR] 0.92, *p* = 0.03), bone recurrence (RR 0.83, *p* = 0.004), and breast cancer mortality (RR 0.91, *p* = 0.04) with a 10-year risk of breast cancer mortality of 16.6% in patients treated with bisphosphonates versus 18.4% in patients who did not receive bisphosphonates. Bisphosphonate therapy was particularly favorable in postmenopausal patients, with an absolute 3.3% reduction in the risk of breast cancer mortality at 10 years and significantly improved bone recurrence rates (RR 0.72, *p* = 0.002). While the benefit seen was independent of estrogen receptor status, tumor grade, axillary nodal status, and type of adjuvant therapy employed, given that the absolute benefit was small, this approach should be used in patients at higher risk of recurrence. According to the guidelines from the American Society of Clinical Oncology (ASCO) and Cancer Care Ontario (CCO), the same criteria used to decide that a patient is a candidate for adjuvant systemic therapy may also apply when deciding on bisphosphonate use [44••].

Also notable from this meta-analysis was that more intense schedules of bisphosphonate therapy, as in the AZURE trial where zoledronic acid was administered every 3–4 weeks for the initial 6 months, were not more efficacious than a more conservative 6-month dosing schedule (more intensive schedule RR 0.84 versus less intensive schedule RR 0.75). Additionally, the specific bisphosphonate used did not significantly affect outcomes, except for pamidronate that was associated with a RR of 1.17 in the 953 patients studied. The ongoing Southwest Oncology Group (SWOG) S0307 trial is also assessing outcomes among patients with early breast cancer treated with clodronate, ibandronate, and zoledronic acid. Preliminary evidence suggests a similar 5-year DFS (88%) and OS (93%) benefit regardless of agent [45]. In current clinical practice, IV zoledronic acid is the preferred agent due to patient compliance (once every 6-month IV dosing) coupled with robust clinical data.

In June of 2017, a joint practice guideline from ASCO and CCO was published which recommended adjuvant IV zoledronic acid in appropriate postmenopausal patients, but noted that additional research was needed to clarify the duration, dose, and dosing interval of bone-modifying agent therapy [44••]. At the 2017 San Antonio Breast Cancer Symposium, preliminary results from the SUCCESS trial were reported, showing no benefit for 5 years of extended IV zoledronic acid therapy versus 2 years [46]. Zoledronic acid was given every 3 months for the first 2 years, and every 6 months thereafter for patients randomized to receive 5 years of therapy. The trial included 3754 women with high-risk early breast cancer, and after a median follow-up of 3 years, DFS and OS were similar (90 and 95%, respectively). However, because the number of events was low, the decision to proceed with less than 5 years of adjuvant bisphosphonate therapy should be tailored to the patient’s risk profile.

While the use of RANK ligand inhibitors has not been fully validated in this setting, the results from two trials evaluating adjuvant denosumab and potential anticancer outcomes were recently presented. The Austrian Breast and Colorectal Cancer Study Group (ABCSG)-18 trial enrolled 3425 postmenopausal patients with early-stage breast cancer receiving treatment with AIs. Patients were randomized 1:1 to receive denosumab 60 mg subcutaneously (SQ) every 6 months or placebo during AI therapy for up to 5 years. After a median follow-up of 72 months, the secondary endpoint of disease-free survival (DFS) was significantly improved in the denosumab arm (80.6% at 8 years compared to 77.5% in the placebo group) [47]. Interestingly, the biggest reduction was observed in second primary, non-breast invasive cancers. Although there was no difference in adverse events, we are awaiting long-term follow-up data and overall survival (OS) signals. The D-CARE trial assessed a more intensive adjuvant denosumab regimen (120 mg SQ monthly for six doses then every 3 months for up to 5 years compared to placebo) in high-risk patients regardless of menopausal status, with over 90% of study participants having node-positive disease. The study terminated early in 2018 following a primary analysis that demonstrated the trial did not meet its primary endpoint of bone metastasis-free survival, with no improvements in the postmenopausal cohort either [48]. Investigators also determined 5.4% of patients treated with denosumab developed osteonecrosis of the jaw (ONJ) compared to 0.2% of patients on placebo, and 0.4% of patients on denosumab experienced an atypical femoral fracture. With conflicting outcomes and immature survival data, RANK ligand inhibitor therapy is not endorsed by the national guidelines in the adjuvant setting.

## Bone-Modifying Agent Therapy in Metastatic Breast Cancer with Bone Involvement

It is estimated that approximately 20–30% of initial early-stage breast cancers will become metastatic [49, 50] and 6–10% of breast cancers are diagnosed as de novo metastatic disease [51, 52]. Furthermore, 70% of metastatic breast cancer patients have bone involvement, and two thirds of these patients will suffer from a skeletal-related event (SRE) [1]. Skeletal-related events include pathologic fracture, the need for palliative radiation to reduce pain, orthopedic intervention for impending fracture, hypercalcemia, or spinal cord compression.

Patients with metastatic breast cancer and evidence of bone metastases, regardless of menopausal status, should receive a bone-modifying agent to help prevent SREs following the results of multiple randomized clinical trials that confirmed a benefit for this indication (Table 1). According to the ASCO guidelines, bone-modifying agents are recommended for patients with evidence of bone destruction on plain radiographs. In patients with normal plain radiographs and abnormal bone scan, it is reasonable to start bone-modifying agents in the presence of an abnormal CT scan or MRI showing bone destruction. However, this is not recommended for women with only abnormal bone scan but without evidence of bone destruction on radiographs, CT scans, or MRI outside of a clinical trial. Different from bisphosphonate use in the adjuvant setting, there is no survival advantage [55]. Zoledronic acid is favored over pamidronate, due to ease of infusion (15 min every 12 weeks versus 120 min every 4 weeks) and a trend toward decreased long-term risk of SREs [56].

**Table 1:** Selected bone-modifying agent studies

Study	*N*	Treatment	Menopausal status	Primary endpoints	Results
Adjuvant					
Bisphosphonates					
Delmas et al. 1997 [26]	53	Risedronate 30 mg daily for 2 weeks every 12 weeks for 2 years (vs placebo)	Premenopausal (enrolled if menses ceased after fchemotherapy)	Change in lumbar spine and proximal femur BMD at 24 months	+ 2.5% difference in lumbar spine BMD, + 2.6% proximal femur BMD at 2 years compared to placebo
Saarto et al. 1997 [27]	148	Clodronate PO 1600 mg daily for 2 years (vs placebo)	Premenopausal	Change in lumbar spine and femoral neck BMD at 24 months	− 2.2% change in lumbar spine BMD, + 0.9% change in femoral neck BMD at 2 years (compared with − 5.9%, − 2.0% with placebo)
ARIBON 2008 [28]	131	Ibandronate 150 mg monthly for 2 years (vs placebo)	Postmenopausal	Change in lumbar spine and hip BMD at 24 months	+ 2.98% change in lumbar spine BMD, + 0.6% change in hip BMD at 2 years (compared with − 3.22%, − 3.9% with placebo)
Diel et al. 2008 [35]	302	Clodronate PO 1600 mg daily for 2 years (vs placebo)	Pre and postmenopausal	Incidence of distant metastases, metastasis-free interval, OS	OS 79.6 vs 59.3% (*p* = 0.04); no significant improvements in incidence of metastases or metastasis-free interval
ABCSG-12 2011 [39]	1803	Zoledronic acid IV 4 mg q6months for 3 years with OFS (vs placebo)	Premenopausal	DFS	Improved DFS (HR 0.60)
CALGB 79809 2011 [29]	439	Zoledronic acid IV 4 mg q3months for 2 years (vs delayed initiation)	Premenopausal	Change in lumbar spine BMD at 12 months	+ 1.0% change in lumbar spine BMD vs − 0.5% with delayed treatment at 3 years
NSABP B-34 2012 [36]	3311	Clodronate PO 1600 mg daily for 3 years (vs placebo)	Pre and postmenopausal	DFS	No differences in DFS (HR 0.91)
Z-FAST 2012 [30]	602	Zoledronic acid IV 4 mg q6months for 5 years (vs delayed initiation)	Postmenopausal	Change in lumbar spine BMD at 12 months	+ 8.9% change in lumbar spine BMD vs + 6.7% with delayed treatment
GAIN 2013 [37]	3023	Ibandronate PO 50 mg daily for 2 years (vs placebo), with OFS if premenopausal after chemotherapy	Pre and postmenopausal	DFS	No differences in DFS (HR 0.95)
ZO-FAST 2013 [31]	1065	Zoledronic acid IV 4 mg q6months for 5 years (vs delayed initiation)	Postmenopausal	Change in lumbar spine BMD at 12 months	+ 4.3% change in lumbar spine BMD vs − 5.4% with delayed treatment
AZURE 2014 [41]	3360	Zoledronic acid IV 4 mg q6months for 5 years (vs placebo)	Pre and postmenopausal	DFS	No differences in DFS (HR 0.94)
SWOG S0307 2015 [45]	6097	Clodronate PO 1600 mg daily, ibandronate PO 50 mg daily, or IV zolendronic acid IV 4 mg q3months for 2.5 years	Pre and postmenopausal	DFS	5-year DFS similar: 88% (clodronate and zoledronic acid), 87% (ibandronate)
SUCCESS 2017 [46]	3754	Zoledronic acid IV 4 mg q3-6 months for 5 years vs q3months for 2 years	Pre and postmenopausal	DFS	No differences in DFS (HR 0.97)
RANK ligand inhibitors					
ABCSG-18 2015 [47]	3425	Denosumab 60 mg SQ q6months while on AI	Postmenopausal	Time to first clinical fracture	Improved fracture rate (HR 0.5)
D-CARE 2018 [48]	4509	Denosumab 120 mg SQ q3months for 5 years	Pre and postmenopausal	Bone metastasis-free survival	No difference in bone metastasis-free survival (HR 0.97)
METASTATIC					
Bisphosphonates					
ZOOM 2013 [59]	425	Zoledronic acid IV 4 mg q4weeks vs q12weeks (after 1 year q4week treatment)	Pre and postmenopausal	Overall skeletal morbidity rate	Skeletal morbidity rate 0.26 (12 weeks) vs 0.22 (4 weeks), non-inferior
OPTIMIZE-2 2017 [60]	416	Zoledronic acid IV 4 mg q4weeks vs q12weeks (after > 9 pre-enrollment doses)	Pre and postmenopausal	SRE rate	1 years SRE rate 23.2% (12 weeks) vs 22% (4 weeks), non-inferior
CALGB 70604 2017 [58]	855	Zoledronic acid IV 4 mg q4weeks vs q12weeks for 2 years	Pre and postmenopausal	SRE rate	2 years SRE rate 27% (4 weeks) vs 29% (12 weeks), non-inferior
RANK ligand inhibitors					
Lipton et al. 2008 [53]	255	5 denosumab regimens (q4week 30 mg/120 mg/180 mg, q12week 60 mg/180 mg) vs IV bisphosphonate q4weeks	Pre and postmenopausal	SRE rate	SRE rate 12% (denosumab) vs 16% (bisphosphonate)
Fizazi et al. 2009 [54]	111	Denosumab SQ 180 mg q4weeks or q12weeks (vs IV bisphosphonate q4weeks) for 25wks	Pre and postmenopausal	Proportion of patients with uNTx < 50 at week 13	13 weeks uNTx < 50 in 71% (denosumab) vs 29% (bisphosphonate)
Stopeck et al. 2010 [67]	2046	Denosumab 120 mg SQ q4weeks (vs IV bisphosphonate q4weeks)	Pre and postmenopausal	SRE rate	Median SRE NR (denosumab) vs 26.4 months (IV bisphosphonate) (HR 0.82)

In a recent Cochrane review that summarized nine clinical trials assessing bisphosphonate use versus placebo in patients with osseous metastatic disease (including 2810 breast cancer patients), bisphosphonate therapy reduced the SRE risk by 14% (RR 0.86, *p* = 0.003) [55]. When evaluating zoledronic acid treatment alone, there was a 41% risk reduction in SREs compared to placebo. No study has compared denosumab to placebo for this indication, though denosumab has been compared to bisphosphonate therapy. In a combined analysis of patients with bone metastases from a variety of solid tumors (36% breast), denosumab 4-week dosing was found to be superior to zoledronic acid 4-week dosing, reducing the risk of first SRE by 17% (HR 0.83, *p* = <0.001) and delaying the time to first on-study SRE by a median of 8.21 months [57]. This difference was most pronounced in patients with breast and hormone-refractory prostate cancer. In the recent Cochrane Review, three studies of 2345 breast cancer patients demonstrated that denosumab use was associated with a 22% reduction in the risk of developing a SRE compared with bisphosphonates (RR 0.78, *p* = <0.001) [55]. Two of the included studies assessed 4-week versus 12-week dosing of denosumab, with results favoring 4-week dosing to maintain suppression of bone turnover markers [53, 54]. The phase III clinical trials SAKK 96/12 (ClinicalTrials.gov identifier: NCT02051218) and REaCT-BTA (ClinicalTrials.gov identifier: NCT02721433) are currently ongoing to further assess the efficacy of a 4-week versus 12-week denosumab schedule.

Regarding zoledronic acid dosing schedules, three randomized controlled trials (ZOOM, OPTIMIZE-2, CALGB 70604) have assessed the efficacy of 12-week versus 4-week dosing in preventing SREs. In each trial, similar rates of SREs were observed regardless of the dosing schedule. In the CALGB 70604 trial, 855 bisphosphonate therapy naïve metastatic breast cancer patients were enrolled and received zoledronic acid for 2 years [58]. Higher rates of ONJ and more frequent elevations in baseline creatinine were appreciated in the 4-week dosing arm (2% versus 1%, *p* = 0.10; 19.9% versus 15.5%, *p* = 0.02), whereas rates of SREs were similar (27% with 4-week dosing versus 29%). In the ZOOM and OPTIMIZE-2 trials, approximately 400 patients were enrolled in each trial to receive zoledronic acid every 4 weeks for 1 year, and then were randomized to receive either 4-week or 12-week dosing [59, 60]. Both trials concluded that 12-week dosing was non-inferior (ZOOM SRE rate 26% with 12-week dosing versus 22%; OPTIMIZE-2 SRE rate 23.2% with 12-week dosing versus 22%). Additionally, in a meta-analysis by Ibrahim et al. evaluating five studies that compared a 4-week dosing schedule to 12-week of pamidronate, or zoledronic acid, the 4-week dosing schedule led to a comparable SRE risk (RR 0.90) with higher risks of ONJ (RR 0.83) [61]. Taken together, a 12-week dosing schedule of zoledronic acid is preferred.

If an SRE occurs despite zoledronic acid 4 mg IV every 12 weeks or denosumab 120 mg SQ every 4 weeks, treatment should continue as the patient remains at risk for subsequent SREs. However, one might consider transitioning therapy to a more potent bisphosphonate or from a bisphosphonate to denosumab. Several clinical studies have suggested an improved response with this approach [54, 62, 63].

Regarding the duration of treatment with bone-modifying agents for metastatic breast cancer, the ASCO guidelines recommend indefinite use until evidence of substantial decline in a patient’s general performance status [64••]. However, there is emphasis on the need to weigh the potential benefits and harms of therapy when considering long-term use of these agents.

The OPTIMIZE-2 trial attempted to analyze duration with a discontinuation of zoledronic acid arm after 1 year of therapy; however, accrual was poor. It may be reasonable to consider discontinuation of bone-modifying agents in patients with stable osseous metastatic disease after 3 to 5 years of use, weighing the risks of continued therapy in terms of ONJ and detrimental bone remodeling against likely modest additional benefits.

## Considerations Prior to Treatment Initiation

Prior to initiation of a bone-modifying agent for treatment-related BMD loss, adjuvant therapy in postmenopausal patients, or to reduce SREs in patients with osseous metastatic disease, the provider should ensure that the patient has undergone appropriate dental clearance and laboratory evaluations. Additionally, patients should be counseled regarding side effects associated with treatment. Notably, a flu-like prodrome (fever and myalgias) may occur in up to 55% of patients [65], typically within 24 h of the infusion. Subsequent pre-treatment with acetaminophen often ameliorates symptoms. Hypocalcemia may develop, particularly on denosumab therapy, and patients should take supplemental calcium and vitamin D during the duration of treatment with a bone-modifying agent.

### Dental Clearance

Bone-modifying agents are associated with a risk of ONJ, which carries significant morbidity. The risk of ONJ increases with frequency, dose, and duration of bisphosphonate therapy, with an incidence of approximately 1.3% [66]. According to Stopeck et al., ONJ was more frequent in patients treated with 2–3 years of denosumab versus zoledronic acid (2.0% versus 1.4%, *p* = 0.39), and at 5 years, the cumulative incidence of ONJ was 4.7% for denosumab-treated patients versus 3.5% for zoledronic acid [67]. Conversely, Lipton et al. reported similar rates of ONJ in 5677 patients who received denosumab 120 mg versus zoledronic acid 4 mg every 4 weeks, 1.8% versus 1.3%, respectively [57]. Risk factors for developing ONJ include poor oral hygiene, recent history of dental extraction, use of a dental appliance, preexisting periodontal disease, glucocorticosteroid and/or antiangiogenic agent use, or radiation therapy [65, 66]. Therefore, prior to treatment initiation, all patients should undergo a dental examination and complete all invasive dental procedures as indicated (including extractions and implants). Additionally, patients should be directed to maintain good oral hygiene with regular dental preventative visits for the duration of their bone-modifying agent therapy.

### Laboratory Evaluations

Bisphosphonate use, mainly with pamidronate, is associated with acute kidney injury, and it is dependent on the dose and the duration of administration (with decreased toxicity associated with lower doses and longer infusion times of IV bisphosphonates). Zoledronic acid dose should be reduced when serum creatinine clearance is ≥ 30 and < 60 mL/min. Because the kidneys do not excrete denosumab, it can be safely used in patients with kidney disease. Hypophosphatemia, hypocalcemia, and hypo or hypermagnesemia may also be appreciated on laboratory evaluations. Hypocalcemia is more common in patients treated with denosumab.

## Recommendations

1. Adjuvant bisphosphonate therapy should be considered for patients with early breast cancer that are candidates for systemic therapy, either postmenopausal or premenopausal patients receiving ovarian function suppression
  Associated with a 3.3% absolute risk reduction in breast cancer mortality.
  Benefit independent of estrogen receptor status and axillary nodal status.
  Efficacy of different bisphosphonates appears to be similar, though IV zoledronic acid or oral clodronate are preferred.
  Limited data for denosumab for this indication.
2. Bone-modifying agents (denosumab or bisphosphonates) are recommended for all metastatic breast cancer patients with bone involvement as previously defined
  Denosumab may be superior to zoledronic acid, though it is associated with increased patient inconvenience (4-week dosing, financial toxicity). Decisions regarding which agent to pursue should take into account patient preference.
  When zoledronic acid is prescribed for this indication, every 12-week dosing is preferred to every 4-week dosing.
  If an SRE occurs on bone-modifying agent therapy, the patient should continue to receive treatment but with a different agent.
  For patients with stable disease, it is reasonable to consider discontinuation of therapy after 5 years, with risks of continued use potentially outweighing further benefit.
3. All patients should undergo dental evaluation and laboratory evaluations including a mineral panel and renal function prior to bone-modifying agent treatment initiation.
4. Routine prophylactic bisphosphonate or denosumab use for BMD protection is not recommended for all patients with early-stage, low-risk breast cancer receiving chemotherapy and/or aromatase inhibitors.
